# Measurements of Rotational Events Generated by Artificial Explosions and External Excitations Using the Optical Fiber Sensors Network

**DOI:** 10.3390/s20216107

**Published:** 2020-10-27

**Authors:** Anna T. Kurzych, Leszek R. Jaroszewicz, Michał Dudek, Jerzy K. Kowalski, Felix Bernauer, Joachim Wassermann, Heiner Igel

**Affiliations:** 1 Institute of Technical Physics, Military University of Technology, 2 gen. S. Kaliskiego St., 00-908 Warsaw, Poland; michal.dudek@wat.edu.pl; 2 Elproma Elektronika Ltd., 13 Szymanowskiego Str., 05-092 Łomianki, Poland; j.kowalski@elpromaelectronics.com; 3 Department of Earth and Environmental Sciences, Ludwig Maximilian University of Munich, Theresienstr. 41, D-80333 Munich, Germany; fbernauer@geophysik.uni-muenchen.de (F.B.); j.wassermann@lmu.de (J.W.); igel@geophysik.uni-muenchen.de (H.I.)

**Keywords:** rotational seismology, optical fiber sensor, detection, Sagnac interferometer

## Abstract

Measurements of artificial events can substantially confirm the data validity of constructed rotational sensors, as well as provide methods for simplifying the measurement process. The above task, especially with international cooperation, can provide full-field measurement results of the target object, which can deliver more significant data and sensor properties. The paper presents vertical rotational velocity recordings gathered during an international experiment that took place at the Geophysical Observatory of the Ludwig Maximilian University of Munich in Fürstenfeldbruck, Germany. Data were obtained during artificial explosions, as well as external excitations induced by a VibroSeis truck. The authors present data recorded by two prototypes of optical fiber rotational sensors. They have been specially designed for rotational seismology needs and are characterized by a theoretical sensitivity equal to 2 × 10^−8^ rad/s/√Hz and a wide measuring range both in amplitude even up to 10 rad/s, and a frequency from DC to 1000 Hz. Their self-noise investigation during the aforementioned experiment showed that both sensors have precision no worse than 2 × 10^−6^ rad/s/sqrt (Hz) in all desired frequency range from 0.01 to 100 Hz. A down-sampling and a spectral analysis of the recorded signals are also presented. The recorded data and their analysis confirmed the performance and reliability of the applied optical fiber rotational sensors. Moreover, the presented international experiment underlines a special necessity for specifying the sensors’ performance test methodologies in the rotational seismology.

## 1. Introduction

The development of rotational sensors has unlocked new opportunities for researchers to provide a device with the capability to observe and detect real rotational events in the field of rotational seismology (RS). RS is an emerging study of all aspects of rotational motions induced by earthquakes, explosions, and ambient vibrations [1]. It is of interest to several disciplines, including seismology [2], earthquake engineering [3,4], seismotectonic [5], as well as Earth-based detection of Einstein’s gravitation waves [6].

Historically, only translational ground displacements and strain measurements in seismology were recorded. Nevertheless, three rotational components can be significant sources of information about Earth’s inner structure, seismic sources, which are very interesting for engineering purposes [7,8]. At this point, the most important thing is to have an appropriate recording device which fully meets the technical requirements of RS [9,10]. RS requires a wide measuring range, ranging from signals with an amplitude of 10^−8^ rad/s in the case of the teleseismic wave rotation measurement [11,12] up to even a few rad/s for the engineering structure research [4,10]. Thus, the significantly wide measuring range of rotational sensors is an engineering challenge for scientists. Additionally, one should take into consideration a wide frequency bandpass starting from 0.01 Hz to several dozen Hz [10,13].

Generally, accelerometers, geophones, and seismometers (short-period and broadband devices) are inertial sensors that are sensitive to external forces acting along their axis of sensitivity [14]. For such instruments, it is hardly possible to separate the contribution of the rotational motion from the translational motion [15]. Due to this complication, special rotational seismometers are constructed and investigated, which, according to an in-depth analysis presented in [10], can be divided into four groups of instruments. The first group is mechanical rotational sensors, such as TAPS (by Polish Academy of Sciences, Warsaw, Poland) [16] and Rotaphone (by Charles University in Prague, Prague, Czech Republic) [17,18]. These are noncommercial instruments that detect a rotation in an indirect way, and since they are based on inertial sensors, their frequency ranges are too narrow to meet RS requirements, so they should be treated as short-period instruments. The second group is devices operating in a direct way and using different technologies such as MEMS (Micro-Electro-Mechanical Systems)—Horizon (EMCORE, Alhambra, CA, USA) [19] or MHD (Magneto-HydroDynamic)—ARS-14, ARS-16 (Applied Technology Associates, Albuquerque, NM, USA) [20]. These are compact, g-insensitive, and low power instruments. However, they are designed for aircraft or space technology, so generally, they have a different frequency range or a too low dynamic range to meet the requirements of RS. The next devices used in RS are liquid-based sensors with so-called electrochemical transducers—R1, R2 (Eentec, Moscow, Russia) [21]. These compact seismometers are used by different seismological research groups but are characterized by above 20% deviations from the nominal value of a scale factor in temperatures above 20 °C, suggesting that the liquid-based technology still requires improvement [22]. The fourth group is optical rotational seismometers using an optical gyro configuration that operates based on the von Laue-Sagnac effect [23]. They are systems operating in the RLG (Ring-Laser Gyroscope) technology: G-Ring (by Ludwig-Maximilians-Universität München, München, Germany), GEO (by University of Canterbury, Christchurch, New Zealand), ROMY (by European Research Council), as well as FOG (Fiber-Optic Gyroscope) technology blueSeis-3A (iXblue, Saint-Germain-en-Laye, France), SRS-5000 (Optolink, Moscow, Russia), AFORS-1 (by Military University of Technology, Warsaw, Poland), and others—widely described in [10]. Nevertheless, the basic features of systems based on FOGs, such as insensitivity to linear motion, high sensitivity, wide measuring range, and portability, make these systems the most suitable devices for RS, as one can see from the study results of their field applications [24].

However, proper and reliable rotational events monitoring requires appropriate sensors testing and verification. Recordings of the experiments conducted using different excitation sources and different test objects can demonstrate the performance and reliability of the applied sensors. It is also very significant to have an opportunity to compare data provided by at least two sensors mounted in the field. International cooperation concerning this issue is indispensable. In the field of rotational seismology, the International Working Group on Rotational Seismology (IWGoRS) has been established to propagate investigations of rotational motions in seismology. This group organized an experiment entitled “Rotation and strain in Seismology: A comparative Sensor Test” at the Geophysical Observatory Fürstenfeldbruck, Germany, from 18 to 22 November 2019, to enable measurements of rotational effects caused by artificial explosions and vibrations network by various rotational sensors to be conducted. The experiment idea was to collect about 40 different rotational motions, strain, and translation sensors. This paper presents the data from this experiment containing rotational events recordings generated by artificial explosions and external excitations caused by a VibroSeis truck obtained by two interferometric optical fiber sensors constructed by the authors and named Fibre-Optic System for Rotational Events and Phenomena Monitoring (FOSREM). FOSREM uses a technical implementation of the FOG [25] for a rotation motion recording. The most significant attribute of FOSREM is the possibility to measure rotations with a theoretical sensitivity equal to 2 × 10^−8^ rad/s/√Hz. Since it secures the detection rotation rate event up to 10 rad/s at a frequency from DC to 1000 Hz, FOSREM meets all technical requirements for RS fully [10].

The structure of this paper is divided into several separate sections. After the introduction, Section 2 briefly describes the construction and laboratory-measured main parameters of the FOSREM type FOS5. In Section 3, the experiment arrangement with sensors localization and position is described. The main results obtained during the generation of artificial explosions, as well as external excitations, along with discussion, are presented in Section 4. Finally, Section 5 presents short conclusions for the paper.

## 2. Construction of the Applied FOSREM Type FOS5

FOSREM type FOS5 was designed to be applied in broadband seismology, demanding a very wide measuring range. Its technology, as all FOGs, is based on the Sagnac effect [23,26], where the interference of two counterpropagating waves are measured in a closed optical path. When the optical loop is rotating, a phase shift between counterpropagating beams emerges proportionally to the rotation rate component perpendicular to the optical path plane. The effect application in the FOGs makes the devices completely insensitive to translational motions, which is the crucial feature of sensors that are supposed to be applied in the rotational seismology research.

**Figure 1:**
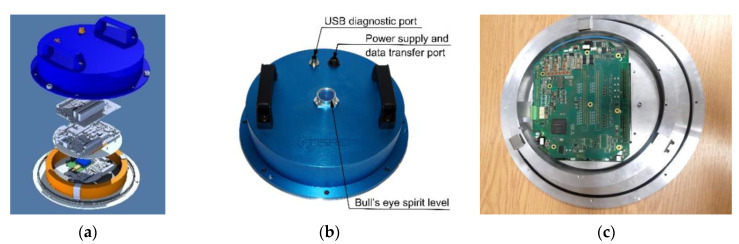
FOS5 construction: (**a**) 3D visualization; yellow ring presents sensor fiber coil inside which on the bottom all optical elements are positioned, together with integrated plate with source, MIOC, and photodetector; next two plates include hardware of applied electronic solution, (**b**) final realization, (**c**) sensors inside; aluminum coil with sensor loop with the plate inside covering the optical part and enabling the assembly of the electronic part. For horizontal FOS5 positioning, it is sensitive for a vertical rotation only.

FOS5 construction is modular (see Figure 1). It consists of two parts: optical and electronic. The optical part is designed and constructed according to the minimum gyro configuration [25], providing reciprocal optical paths for two counterpropagating beams in a fiber loop. It is based on our previous construction [27] and contains a light source—superluminescent diode (Exalos AG, Zürich, Switzerland), isolator (FCA, Niepołomice, Poland), two depolarizers (Phoenix Photonics, Birchington, UK), photodetector APD-1310 (Opotway, Taiwan), coupler (Phoenix Photonics, Birchington, UK), integrated optic chip (MIOC, IdealPhotonics Ltd., Shanghai, China), as well as 5 km of a single optical fiber SMF-28e+ (Corning Inc., New York, NY, USA) as a fiber coil wound as a quadrupole bifilar structure. MIOC is fabricated as a lithium niobate wafer by a high-temperature proton exchange technique [28], and it has two functions. First, it is a Y-type reciprocal coupler that splits beams into two counterpropagating waves equally and then recombines them. Second, its most important function is an electro-optical phase modulation protecting system operating in a closed-loop scheme [25]. Since MIOC is not equipped with a planar polarizers structure, the additional optical fiber polarizer (Phoenix Photonics, Birchington, UK) was applied, which reduces bias instability due to the polarization non-reciprocity.

A new electronic part applies a closed-loop system operation where the Sagnac phase shift is compensated [25]. The original, stipulated (by Elproma Elektronika Ltd., Łomianki, Poland) hardware solution was applied, where the ramp modulation is used to reduce the Sagnac phase shift induced by the rotation in an internal feedback loop. This technology offers high sensor sensitivity together with a wider dynamic range. It consists of the following main modules: SLED driver (Exalos AG, Zürich, Switzerland), four-step modulator, analog amplifier for avalanche photodiode (APD), control based on field-programmable gate array (FPGA), and power with main functionalities, broadly described in [29]. The internal digital processing unit provides a rotation speed value directly in a digital form. Moreover, FOS5 can be controlled fully by the Internet, and downloading data at any time is ensured. FOS5 operates with a 1 ms sampling rate, which secures a 1000 sps data transfer. FOS5, with its diameter equal to 312 mm and height equal to 85 mm, is a compact and integrated device. To apply the sensor in all environmental conditions, it is hermetically sealed and equipped with waterproof connectors meeting the IP67 requirements (see Figure 1b).

**Figure 2:**
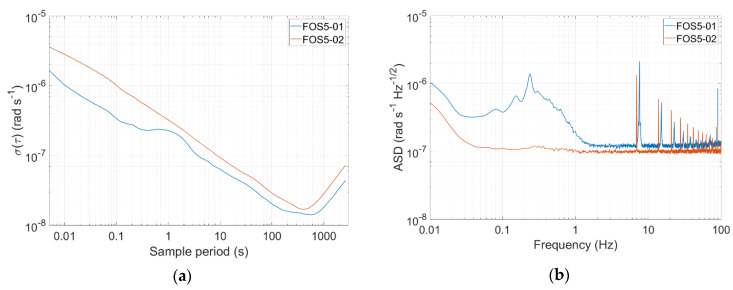
General FOS5-01 and FOS5-02 accuracy investigations: (**a**) Allan variance analysis in Warsaw, Poland (**b**) self-noise amplitude spectral densities recordings at night time 19 November 2019, during the experiment in Furstenfeldbruck.

In the Fürstenfeldbruck field experiment, a set of two FOS5, i.e., FOS5-01 and FOS5-02, has been used. The Allan variance analysis [30,31] revealed the following parameters: for FOS5-01—angle random walk equals 2.16 × 10^−7^ rad/√s, bias instability equals 2.28 × 10^−8^ rad/s, while for FOS5-02—angle random walk equals 3.24 × 10^−7^ rad/√s, and bias instability equals 2.55 × 10^−8^ rad/s (Figure 2a). These values correspond with the theoretical sensitivity of the order of 2 × 10^−8^ rad/s/√Hz. The self-noise investigation during the Fürstenfeldbruck field experiment (see Figure 2b) indicated that the sensors’ amplitude spectral density was no worse than 2 × 10^−6^ rad/s/sqrt (Hz) in the whole investigated frequency range from 0.01 to 100 Hz, and for FOS5-02, it was even at the level of 1 × 10^−7^ rad/s/sqrt (Hz) above 0.03 Hz with the only distinctive narrow peaks at ~7 Hz and its multiplies. The existence of those peaks in the registered self-noise signals and the differences in spectral characteristics between FOS5-01 and FOS5-02 are due to the specific electronics used in the sensors.

## 3. Experiment Description

**Figure 3:**
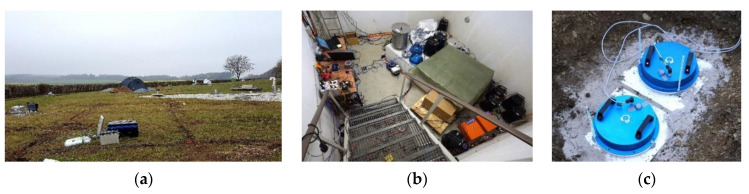
The field of experiment entitled “Rotation and strain in Seismology: A comparative Sensor Test”, Geophysical Observatory Fürstenfeldbruck, Germany: (**a**) general view of the experiment field, (**b**) devices located in the bunker, (**c**) FOS5-01 and FOS5-02 located in the field.

The experiment “Rotation and strain in Seismology: A comparative Sensor Test” was one of a kind due to the number of applied rotational sensors delivered by different research centers. About 40 sensors were placed together in the field (see Figure 3).

One can distinguish the following applied sensors: two blueSeis-3A, ROMY (large 4-component ring laser gyroscope [32]), and three permanent broadband stations (by Ludwig Maximilian University of Munich, Munich, Germany), 80 Channel Geophone system (by ETH, Zürich, Switzerland), three blueSeis-3A (by University of Potsdam, Potsdam, Germany), blueSeis-3A (by Bundesanstalt für Geowissenschaften und Rohstoffe, Hannover, Germany), blueSeis-3A (by ISAE SUPAERO, Toulouse, France) four Rotaphones (by Charles University, Prague, Czech Republic), two Gladiator and three Horizon (by Opole University of Technology, Opole, Poland), four Quadrans, one Octans and several accelerometers (by CEA, Paris, France), giant FOG, blueSeis-3A (iXblue, Saint-Germain-en-Laye, France), giant FOG FARO (Streckeisen GmbH, Zürich, Germany), Distributed Acoustic Sensing cable (DAS, ETH Zurich, Zürich, Switzerland), as well as FOSREMs type FOS3 and FOS5 (by Military University of Technology, Warsaw, Poland).

**Figure 4:**
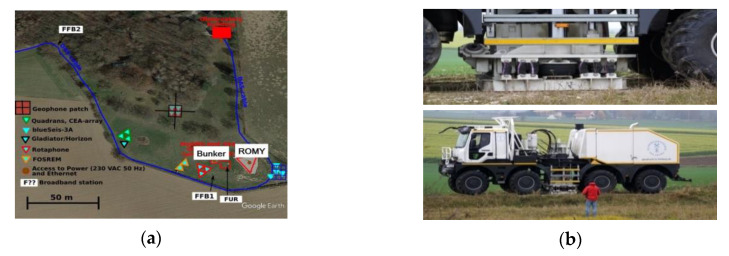
The second part of the Fürstenfeldbruck experiment: (**a**) scheme of sensors localization during the field test (see chart legend for the FOSREM localization), (**b**) a view of the VibroSeis truck applied during the experiment.

The experiment was divided into two parts: artificial explosions with different loads of dynamite within distances from 10 m to 2 km, which were carried out by the Bayrisches Landesamt für Umwelt, Germany, and recording of external excitations generated by a special VibroSeis truck (peak force: 275 kN) provided by TU Bergakademie Freiberg. During the first part, all devices were placed in the seismic bunker (see Figure 3b). The concrete seismic bunker was located on the north side and directly opposite the ROMY installation (ROMY is marked as red triangle in the lower right corner in Figure 4a). It was an underground structure (of about 5 m × 5 m × 3 m), and the floor level is about 6 m below the ground level. The monument in the center of the room (where the FOS5 sensors were installed using three spikes with horizontal positioning by bull’s eye spirit level) was seismically isolated from the rest of the building. In addition, the area where the experiment took place was thermally isolated. During the second part, all FOSREMs were placed in the field shown in Figure 3a, while Figure 4a shows the scheme of sensors’ locations in the field.

Initially, about 0.5 m deep holes were prepared in the ground where 30 × 30-cm square and 12-mm thickness steel plates were hammered into the ground using four 30-cm long pins. Next, the FOS5s were screwed stiffly to the plates by three screws and horizontally positioned using a bull’s eye spirit level (see Figure 3c). Finally, the holes with sensors were heaped with soil. Such an approach was related to the geology of the area, where the ground is terminal Riss moraine (till), as well as to avoid ‘the block rotation’, as shown on the wet sponge, i.e., rocking resonance. There should be no problem with very long waves, but if some short waves appeared, the sensor-plate system could resonate. Thus, it would not show the actual ground surface rocking. Therefore, it is always preferred that the plate be light, so that the applied “sensor + steel plate” system has a higher resonance frequency for rocking on the ground. The photo of the applied VibroSeis truck is shown in Figure 4b. The man in the foreground of the photograph is a reference for understanding the scale of the machine. A typical mass of the VibroSeis machine is about 29,500 kg. A semi-trailer or other truck traveling along the road generates a half-sine wave in the road surface as it passes the point. A VibroSeis truck, with its pre-set mass, is designed to generate full-sine waves. The VibroSeis truck was set to operate for a time period equaling 15 s, with a frequency sweep from about 7 Hz to 120 Hz.

It should be noted that the FOS5s installation during the experiments only allowed registration of vertical rotation. The special interest in obtaining the vertical rotation comes from the fact that only horizontally polarized S-waves (SH-waves) contain a rotational motion signal around the vertical axis. Thus, a vertical rotation sensor can serve as an SH-wave type filter. Furthermore, e.g., in [33], it was shown that it is possible to estimate the local SH-wave phase velocity from joint observations of vertical rotation rate and transverse acceleration at a single point in space. The transverse acceleration *a_T_* is equal to the vertical component of rotation rate ω*_z_* multiplied by twice the local (apparent) horizontal phase velocity *c*: *a_T_* (*x*, *t*) = 2*c* ω*_z_* (*x*, *t*). In other words, under the plane wave assumption, transverse acceleration and rotation rate should have the same waveform, and their amplitudes should scale proportionally to phase velocity depending on wave type (e.g., shear waves, Love waves) and propagation direction [34].

## 4. Results of Recorded Rotational Events Generated by Artificial Explosions and External Excitations Generated by the VibroSeis Truck

**Figure 5:**
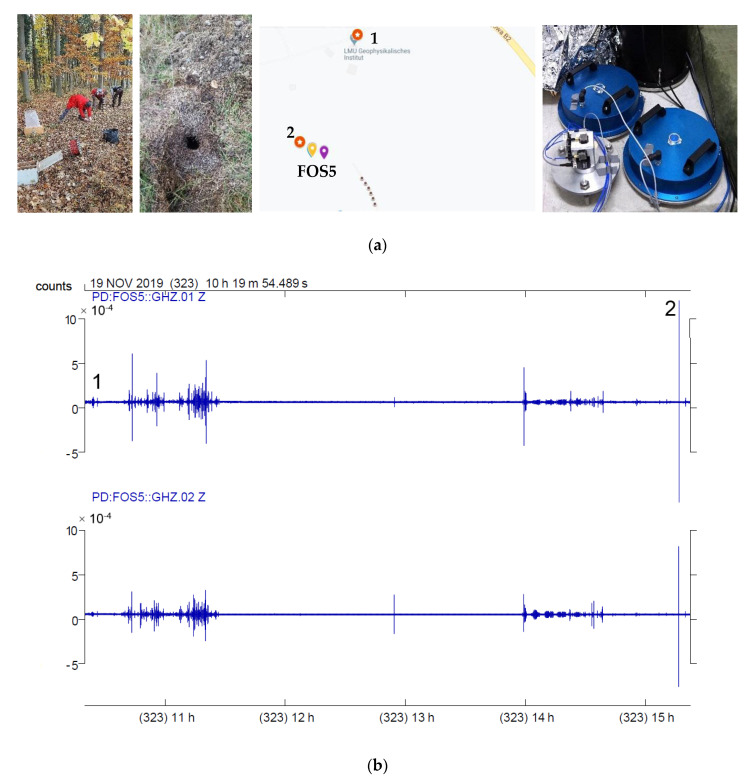
Indication of explosions during the experiment: (**a**) the technical preparation for explosion number 1, the chart with the places of explosions, and FOS5s’ (yellow marker on the map) localization in the bunker (violet marker on the chart); (**b**) the general seismograms for FOS5-01 (top), and FOS5-02 (bottom) which took place on 19 November 2019 with identification of explosion number 1 and 2.

Two explosions took place during the first part of the experiment on 19 November 2019, which differ in the amount of dynamite and distance from sensors located in the bunker (see Figure 5a, Table 1). Figure 5b presents the seismograms of these events for FOS5s prepared in the commercial software SeisGram2Kv8.0 (ALomax Scientific, Mouans-Sartoux, France).

The presented data identify the general proper time of explosions records by FOS5s and the existing rotational motion in the vertical axis (direction of FOS5s detection). Table 1 indicates the parameters of explosions, as well as the signals’ maximum amplitude for a particular event recorded by FOS5-01 and -02, together with a signal energy coefficient calculated numerically using the method of rectangles of the Riemann integral [4]. As shown in Table 1, FOS5s recorded the signal lower maximum amplitude (5.86 × 10^−5^ rad/s (FOS5-01); 3.04 × 10^−5^ rad/s (FOS5-02)) during the explosion with a lower amount of explosive (150 g) and located farther away from FOS5 compared with the signal maximum amplitude (135.78 × 10^−5^ rad/s (FOS5-01); 86.28 × 10^−5^ rad/s (FOS5-02)) during the explosion with the amount of explosive equal to 500 g, as one would have expected. It is evident that FOS5-01 shows higher output signals in comparison with FOS5-02. It was most probably caused by the calibration procedure and should be improved in the future system investigation. However, if we compared the signals’ ratio for two explosions (number 2 and number 1), as a ratio of signal energy coefficient proportional to sensor detected energy, we obtained a similar ratio for the two devices (10.95 and 10.63 for FOS5-01 and FOS5-02, respectively).

**Figure 6:**
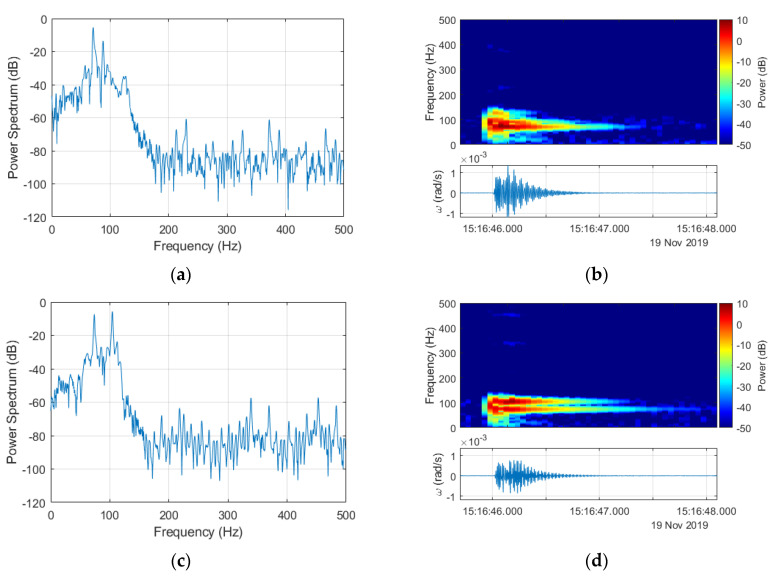
Spectral characteristics of signals recorded by FOS5-01 (**a**,**b**) and FOS5-02 (**c**,**d**) for explosion number 2 registered 19 November 2019 at 15:16 recorded with a fundamental 1-kHz frequency.

Spectral investigation of the recorded signals is presented below in Figure 6, based on explosion number 2 on 19 November 2019, at 15:16 (UTC). The original signals with a 1-kHz sampling rate were cut off frequency at around 120 Hz. It is linked with the characteristics of electronic components of FOS5, where applied electronic filters strongly limited the bandwidth. In addition, the sampling with an original frequency of 1 kHz seemed to be the excessive condition data-wise.

**Figure 7:**
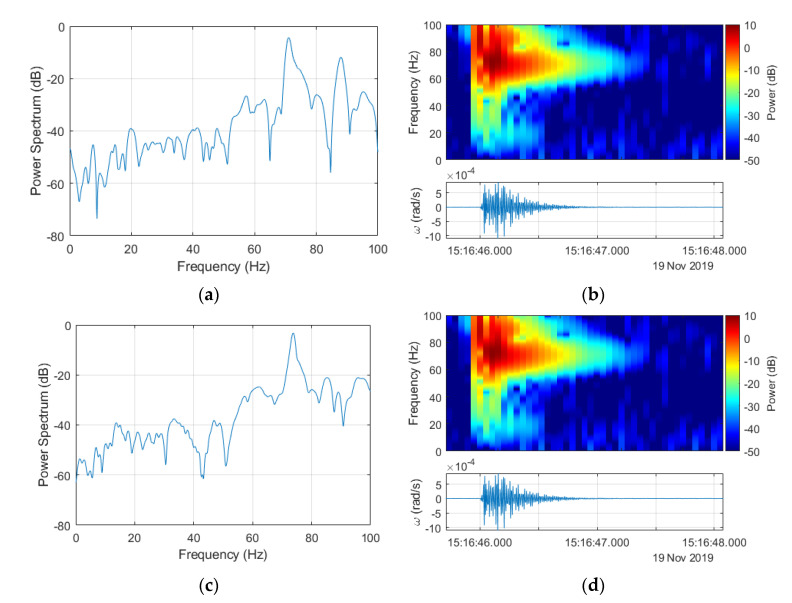
Spectral characteristics from Figure 6 after signals resampling to 200 Hz for FOS5-01 (**a**,**b**) and FOS5-02 (**c**,**d**).

Based on the argument presented above, all further investigations were made with the signals resampled to 200 Hz. The down-sampling from 1 kHz to 200 Hz was preceded by applying an antialiasing filter to the signal using the Kaiser window method. Filter coefficients were also normalized to account for the processing gain of the window. Finally, the filtered signal sample rate was decreased by keeping the first sample and then every 5th sample after the first one. The results of this application to signals obtained for explosion number 2 for both FOS5s are presented in Figure 7. All the power spectra and spectrograms were obtained for signals from FOS5-01 and FOS5-02 (shown as bottom plots in Figure 6b,d, Figure 7b,d and Figure 10b,d) divided by their respective values of standard deviations to normalize both signals according to their noise levels. It is also important to note that the power spectra are shown in Figure 6a,c and Figure 7a,c were calculated for a constant frequency resolution value of 1 Hz, while to obtain spectrograms (Figure 6b,d and Figure 7b,d), the time resolution was set as constant to 0.2 s. Both of those signals were at a constant power level in the frequency range from 0 to 50 Hz with only slight fluctuations, whereas the observed increased power spectral density (PSD) level was between 50 and 100 Hz from the explosion plus every other signal contained in the explosion time window.

**Figure 8:**
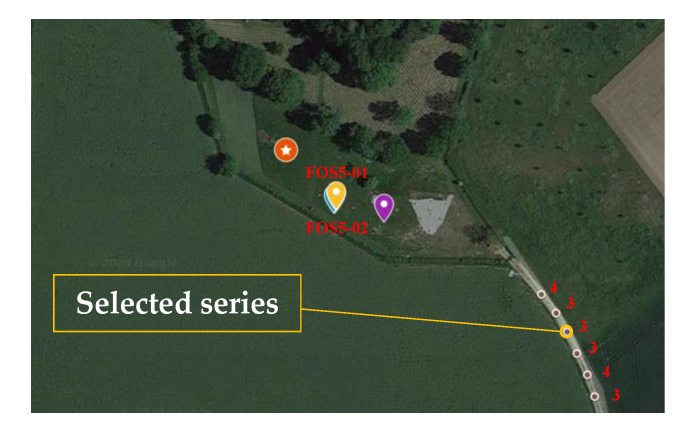
Satellite image with the location of sensors (yellow markers on the chart) and the place of external excitations generated by the VibroSeis truck 21 November 2019, with marking analyzed series.

The second part of the experiment consisted of recordings of external excitations generated by the VibroSeis truck. The experiment took place from 13:44 to 13:54 (UTC) 21 November 2019. The truck stopped six times every 1–2 min to perform excitations, whose characteristics have been described above. The distance between sweeps was equal to 10 m. The distance between FOS5s and the VibroSeis truck operation was in the range from 96 to 138 m (Figure 8). The number of the VibroSeis truck excitations was in the order of 4, 3, 3, 3, 4, 3.

Table 2 shows the individual parameters of the VibroSeis truck operation with the maximum amplitude of signal recorded by FOS5s. The average value of the maximum amplitude during all VibroSeis truck excitations recorded by FOS5s was equal to 2.15 × 10^−5^ ± 0.86 × 10^−5^ (FOS5-01) and 1.57 × 10^−5^ ± 0.31 × 10^−5^ (FOS5-02) rad/s. It can be clearly seen from Table 2 that the applied change in distance between FOS5s and the VibroSeis truck in the range from 96 to 138 m did not change the maximum amplitude of the recorded signal.

**Figure 9:**
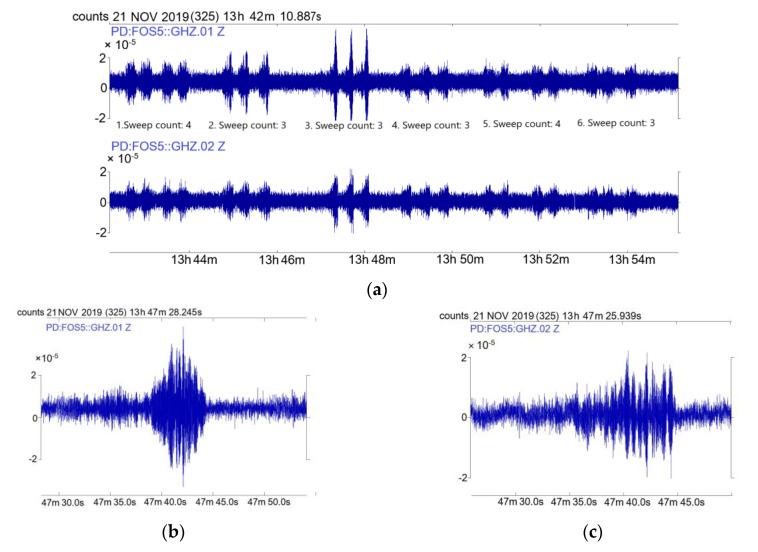
Data recorded during vibrations generated by the VibroSeis truck 21 November 2019: (**a**) the general view of data recorded during all six sweep series by FOS5-01 (top) and FOS5-02 (bottom), (**b**) one sweep plot from Sweep count 3 recorded by FOS5-01, (**c**) one sweep plot from Sweep count 3 recorded by FOS5-02. All data are for the signal resampled to 200 Hz according to the described procedure.

**Figure 10:**
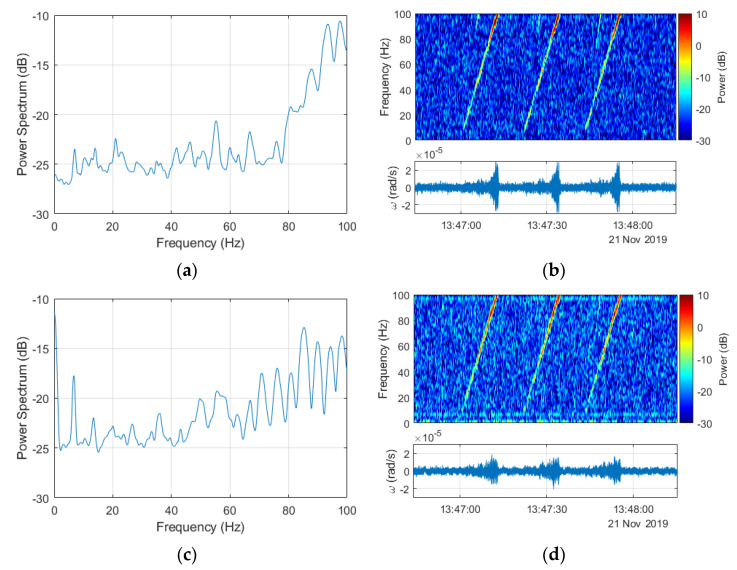
Spectral characteristics of signals recorded during external excitations generated by the VibroSeis truck 21 November 2019 down-sampled to 200 Hz: (**a**) spectrum of the signal presented in Figure 9a recorded by FOS5-01, (**b**) spectral characteristics for the signal recorded by FOS5-01 for sweep count no. 3, (**c**) spectrum of the signal presented in Figure 9a recorded by FOS5-02, (**d**) spectral characteristics for the signal recorded by FOS5-02 for sweep count no. 3.

The plots presented in Figure 9 indicate that both FOS5-01 and FOS5-02 recorded all six sweep changes, and the signals well reflected the amount of the sweep count. The examples of spectral characteristics were prepared according to the previously described procedure, for sweep count: 3 are presented in Figure 10.

A power spectrum for both devices, FOS5-01 and FOS5-02 (left column in Figure 10), was at a constant power level in the range from 0 to 70 Hz with only slight fluctuations with an increase in the range from 70 to 100 Hz. The right column in Figure 10 presents spectrograms of signals from sweep count series 3, obtained by FOS5-01 and FOS5-02, respectively. It is also important to note that the power spectra shown in Figure 10a,c were calculated for a constant frequency resolution value of 1 Hz, while the obtained spectrograms (Figure 10b,d) and time resolution was set as constant to 1 s. Both spectrograms represent changes in a registered wave frequency over time. From the obtained figures, it is evident that the frequency of simulated shock waves started at around 10 Hz and then linearly increased up to 100 Hz and possibly even further until their extinction. An increase in the frequency above around 70–80 Hz corresponded with an increase in the signal amplitudes, which was especially evident for FOS5-01. From the above, we can conclude that recorded FOSs signals indicated the same range of frequency generated by the VibroSeis truck (7 Hz to 120 Hz). Some electronic buzz, not identified yet, was evident, especially when comparing the power spectrum and spectrogram of signals from FOS5-02, at around 7 Hz and 97 Hz. In addition, the peak in the FOS5-02 (Figure 10c,d) power spectrum at around 0 Hz suggests a constant shift of the signal, which was not visible in the seismogram and may result from slow-varying heat instabilities.

## 5. Conclusions

Rotational seismometers have great potential in many applications, such as seismic tomography, scattering analysis, ocean-bottom observations, volcanology, exploration, or structural engineering. However, as was outlined in the introduction, they are still in a development state, so international cooperation employing different rotational sensors during one experiment is highly welcomed. Significantly, the field measurements are important to standardize the sensors’ performance test methodologies. This paper presents the actions currently being undertaken to bring the benefits of an integrated international experiment dealing with the rotational seismology sensors technology which took place at the LMU Geophysical Observatory in Germany. It provided the opportunity to apply various rotational sensors in one place exposed to artificial vibrations. The authors presented the data obtained by two interferometric optical fiber sensors FOS5-01 and -02. The laboratory results indicated that those devices are characterized by parameters meeting all technical requirements for rotational seismology. The Allan variance analysis revealed that they could register a signal at the order of 10^−7^ rad/s, whereas a self-noise investigation during the experiment identified the precision of the sensor not to be worse than 2 × 10^−6^ rad/s/sqrt (Hz) in the frequency band between 0.01 and 100 Hz.

The results presented in this paper indicated that an external explosion and external excitations by the special VibroSeis truck could generate vertical rotational events because they were recorded by FOS5s, which are sensitive only to such rotational motion. The rotational events generated in the presented experiment were propagated in Riss moraine ground with a good frequency transfer. To adjust the signals to the characteristics of the generated excitations, there were down-samplings from 1 kHz to 200 Hz preceded by applying an antialiasing filter to the signal using the Kaiser window method.

The data obtained during the external explosions by FOS5-01 and -02 showed good time correlation with the time of the explosions. Moreover, the power spectra of signals registered during the VibroSeis truck operation confirmed that both FOS5s registered data containing the generated frequency components of an induced signal. Unfortunately, the registered different self-noise characteristics of FOS5-01 and -02 indicated the main source of differences in signals registered by these two sensors. The growing noise level for the middle range of frequencies in FOS5-01 and the existence of additional peaks in the higher frequencies for both sensors were mostly due to the specific electronics used in FOS5s. This issue will be one of the main subjects in further FOS5s investigation and improvement. Nevertheless, the signals from both sensors correlated, especially for a given frequency range where the power level increases.

The conducted measurements point out the importance of undertaking sensors field testing where uniformity in sensor mounting and as well ground influence has to be taken into consideration. The performed spectral analysis underlines the importance of sensor frequency range and signal filtering.

## Figures and Tables

**Table 1:** Parameters of artificial explosions with the maximum amplitude of signal recorded by FOS5s.

Number of Explosions	Date	Time (UTC)	Amount of Explosive (g)	Distance from FOS5s (m)	Maximum Signal Amplitude (rad/s) for FOS5-01/02	Signal Energy Coefficient (rad) FOS5-01/02
1	19 November 2019	10:26	150	220	5.86 × 10^−5^/3.04 × 10^−5^	1.71 × 10^−5^/1.9 × 10^−5^
2	19 November 2019	15:16	500	52	135.78 × 10^−5^/86.28 × 10^−5^	18.72 × 10^−5^/13.71 × 10^−5^

**Table 2:** Parameters of VibroSeis truck work with the maximum amplitude of signal recorded by FOS5s.

Data	Number of Series	Time (UTC)	Distance from FOS5s (m)	Signal Maximum Amplitude (rad/s) FOS5-01/02
21 November 2019	1	13:44	96	1.77 × 10^−5^/1.66 × 10^−5^
	2	13:45	105	2.52 × 10^−5^/1.54 × 10^−5^
	3	13:48	113	3.86 × 10^−5^/2.20 × 10^−5^
	4	13:49	121	1.58 × 10^−5^/1.32 × 10^−5^
	5	13:52	130	1.56 × 10^−5^/1.44 × 10^−5^
	6	13:54	138	1.41 × 10^−5^/1.25 × 10^−5^
				Average: 2.15 × 10^−5^ ± 0.86 × 10^−5^/1.57 × 10^−5^ ± 0.31 × 10^−5^
